# A Signature of Four Circulating microRNAs as Potential Biomarkers for Diagnosing Early-Stage Breast Cancer

**DOI:** 10.3390/ijms22116121

**Published:** 2021-06-06

**Authors:** Maha M. Itani, Farah J. Nassar, Arafat H. Tfayli, Rabih S. Talhouk, Ghada K. Chamandi, Abdul Rahman S. Itani, Joelle Makoukji, Rose-Mary N. Boustany, Lifang Hou, Nathalie K. Zgheib, Rihab R. Nasr

**Affiliations:** 1 Department of Anatomy, Cell Biology and Physiological Sciences, Faculty of Medicine, American University of Beirut, Beirut 1107 2020, Lebanon; mmi40@mail.aub.edu (M.M.I.); gc21@aub.edu.lb (G.K.C.); abed.s.itani@gmail.com (A.R.S.I.); 2 Department of Internal Medicine, Faculty of Medicine, American University of Beirut, Beirut 1107 2020, Lebanon; fjn00@mail.aub.edu (F.J.N.); at35@aub.edu.lb (A.H.T.); 3 Department of Biology, Faculty of Arts and Sciences, American University of Beirut, Beirut 1107 2020, Lebanon; rtalhouk@aub.edu.lb; 4 Pathophysiology of Breast Cancer Team, INSERM U976, HIPI, Université de Paris, 75010 Paris, France; 5 Department of Biochemistry and Molecular Genetics, Faculty of Medicine, American University of Beirut, Beirut 1107 2020, Lebanon; jm53@aub.edu.lb (J.M.); rb50@aub.edu.lb (R.-M.N.B.); 6 Center of Global Oncology, Northwestern University, Chicago, IL 60611, USA; l-hou@northwestern.edu; 7 Department of Pharmacology and Toxicology, Faculty of Medicine, American University of Beirut, Beirut 1107 2020, Lebanon

**Keywords:** microRNA, breast cancer, diagnosis, circulating biomarkers, liquid biopsy, ROC curve, early stage

## Abstract

Breast cancer (BC) is the most predominant type of cancer among women. The aim of this study is to find new biomarkers that can help in early detection of BC, especially for those who are too young to be screened using mammography as per guidelines. Using microRNA microarray, we previously showed dysregulation of 74 microRNAs in tumors from early BC patients as compared with normal adjacent tissues, which we were interested in studying in blood circulation. In this study, we investigated the expression of 12 microRNA (miR-21/miR-155/miR-23a/miR-130a/miR-145/miR-425-5p/miR-139-5p/miR-451/miR-195/miR-125b/miR-100, and miR-182) in the plasma of 41 newly diagnosed Lebanese BC patients with early invasive ductal carcinoma as compared with 32 healthy controls. Total RNA was extracted from plasma, and expression levels of miRNA of interest were measured using RT-qPCR followed by statistical analysis; miR-21, miR-155, miR-23a, miR-130a, miR-145, miR-425-5p, and miR-139-5p were significantly upregulated and miR-451 was significantly downregulated, in the plasma of BC patients as compared with healthy controls. The positively correlated miR-23a, miR-21, and miR-130a had a high diagnostic accuracy (86%). Importantly, the combination of miR-145/miR-425-5p/miR-139-5p/miR-130a scored the highest diagnostic accuracy of 95% with AUC = 0.97 (sensitivity 97% and specificity 91%). MicroRNAs are promising non-invasive diagnostic biomarkers for early-stage BC with the panel of miR-145/miR-425-5p/miR-139-5p/miR-130a having the highest diagnostic accuracy.

## 1. Introduction

Breast cancer (BC) is a commonly diagnosed type of cancer worldwide, and the leading cause of cancer-related deaths among women [1]. According to GLOBOCAN 2018, Lebanon ranked sixth in the estimated age-standardized BC incidence rate among women, and it was estimated to cause 920 deaths, and therefore BC is the top cancer-related mortality in females [2]. Although mammography is the gold standard for BC detection, it is associated with pain, anxiety, and radiation exposure [3]. Its efficiency is restricted by dense breasts [4]. Most importantly, mammography is not recommended for individuals <40 years which constitutes 17.33% of Lebanese BC patients [2]. Thus, it is of great importance to find novel reliable, sensitive, and minimally invasive biomarkers which could help in early detection of BC.

MicroRNAs (miRNAs) are small (~22 nucleotides), single stranded, non-coding RNA molecules [5]. They have a fundamental role in several physiological and pathophysiological cellular processes. MicroRNA regulates gene expression post transcriptionally either through silencing or degrading mRNA molecules involved in oncogenic or tumor-suppressor signaling pathways [6]. Circulating microRNAs are present in body fluids either in the form of free, unbound miRNAs, miRNAs bound to proteins, or membrane encapsulated miRNAs including those in microvesicles and exosomes [7]. Their characteristics of stability, ease of detection, and minimal invasiveness solidify their utility as promising diagnostic, prognostic, and predictive biomarkers for BC [8].

Previous studies involving microRNA microarray profiling have identified 74 differentially expressed miRNAs in BC tissues as compared with normal adjacent tissues from Lebanese patients with early-stage BC [9,10]. Among these dysregulated miRNAs, miR-21, miR-155, miR-425-5p, and miR-182 were significantly upregulated, while miR-130a, miR-195, miR-451, miR-139-5p, miR-145, miR-125b, and miR-100 were significantly downregulated in tumor tissue of early-stage BC patients [10]. Emerging studies have reported positive correlations between body fluid miRNAs and tissue miRNA levels, which indicated a potential role for circulating miRNAs as biomarkers to monitor corresponding human cancers, including BC [11]. Additionally, all of the above-mentioned miRNA molecules, except miR-100 and miR-23a, have demonstrated differential expression in the blood circulation of BC patients as compared with normal controls [8,12,13]. Here, the value of the above extensively studied 12 miRNAs (miR-21, miR-155, miR-23a, miR-130a, miR-145, miR-425-5p, miR-139-5p, miR-451, miR-195, miR-125b, miR-100, and miR-182) are investigated as novel diagnostic circulating biomarkers for detecting early-stage BC in Lebanese patients [8,14].

## 2. Results

### 2.1. Patient Characteristics

The clinical and pathological data of the 41 recruited BC patients are shown in Table 1. The average age of the BC participants at diagnosis was 53 years, ranging from 30 to 84 years. Healthy control blood samples were collected from healthy women donors with an average age of 34.4 years, ranging from 21 to 60 years. Pathologically, all cases were of the invasive ductal carcinoma (IDC) BC type with positive estrogen and progesterone receptor (ER and PR) status; 63.4% of the cases were HER-2 positive and 41.5% were premenopausal at the time of diagnosis. More than half of the participants had a tumor size ≤2 cm (65.9%) with no lymph node involvement (61%). All patients were free of distant metastases (M0) and therefore were, referred to as early-stage BC patients.

### 2.2. MiRNA Expression in the Plasma of Breast Cancer Patients as Compared with Healthy Subjects

**Figure 1:**
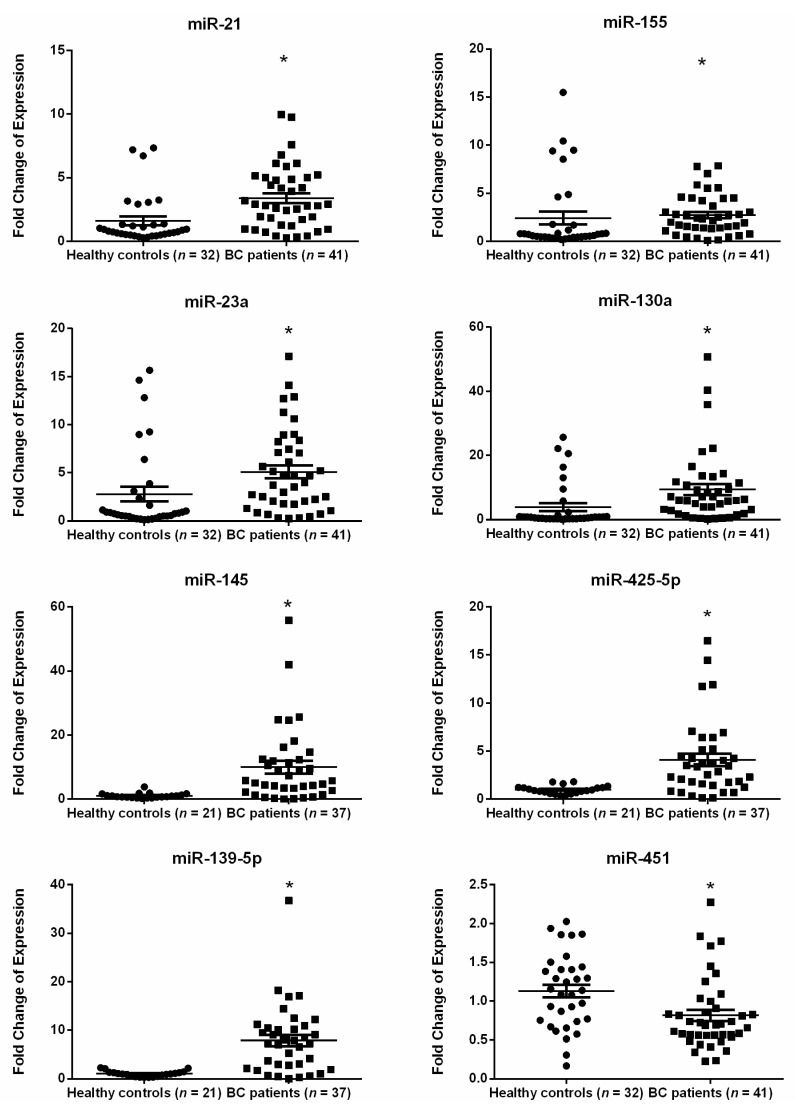
Fold change of expression for the significantly dysregulated miRNA in the plasma of Lebanese women with early-stage BC as compared with healthy controls. The plots represent the mean (middle line) and the standard error of mean (error bars). * Denotes *p* < 0.05 according to Wilcoxon’s signed-rank test.

Depending on the availability of plasma samples, miRNA expression detection occurred in different sample sizes. RT-qPCR showed significant upregulation in the relative expression of plasma miR-21, miR-155, miR-23a, miR-130a, miR-145, miR-425-5p, and miR-139-5p (*p* < 0.0001 for each miRNA) in early-stage BC patients, whereas a significant downregulation was demonstrated in expression of miR-451 (*p*-value = 0.0049) as compared with healthy subjects (Figure 1). In contrast, miR-195, miR-125b, miR-100, and miR-182 were non-significantly deregulated in plasma of early-stage BC patients (Supplementary Material Figure S1).

### 2.3. Expression of miRNAs in Early Stage BC Patients with Different Clinicopathological Features

**Figure 2:**
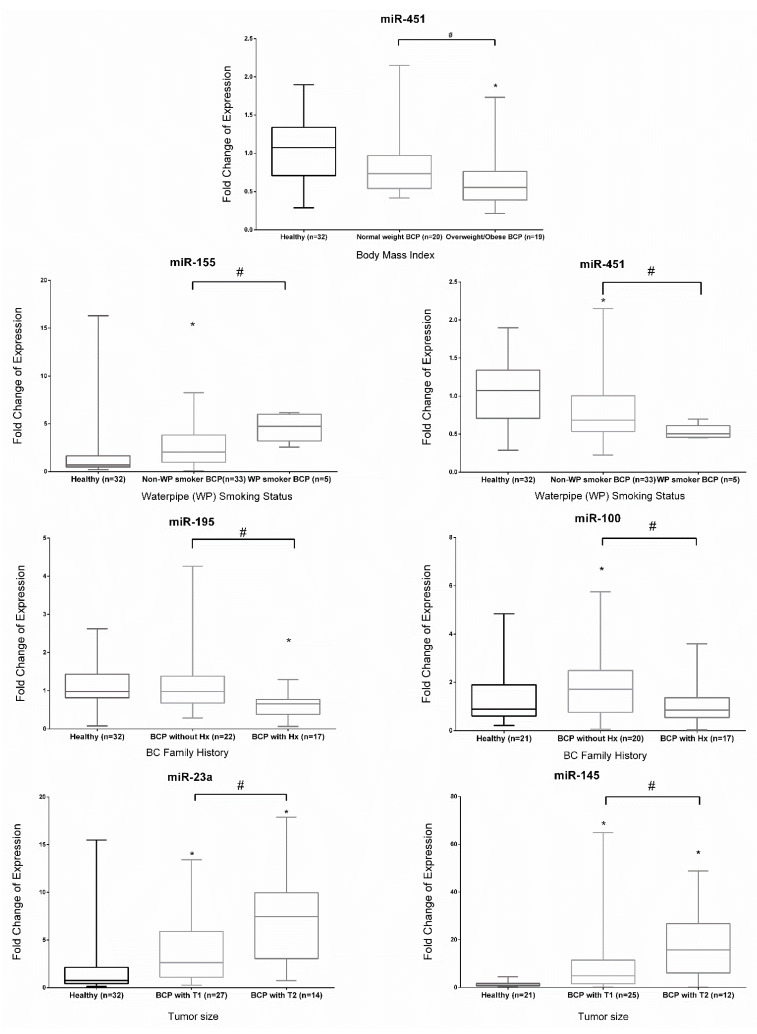
Fold change of expression for specific miRNA in subgroups of BC patients as compared to healthy controls. The top, middle, and bottom lines of the boxplots represent the 25% percentile, median, and 75% percentile, respectively. The whiskers represent minimum and maximum. * Denotes *p* < 0.05, using Wilcoxon’s signed-rank test to compare the significance of the fold change expression of miRNA in BC to 1. # Denotes *p* < 0.05 using Mann–Whitney U test to compare the significant dysregulation of the miRNA within two or more clinicopathological subgroups. BCP, breast cancer patients; T1, ≤2 cm; T2, >2 cm.

The fold change of expression of each miRNA studied within different clinicopathological subgroups of early-stage BC patients is illustrated in Supplementary Material Table S1 and Figure 2. Subgrouping BC patients according to body mass index (BMI), miR-451 showed a significant decreased expression in plasma of obese/overweight (BMI > 25 kg/m^2^) vs. normal weight (BMI > 20 and < 24.9 kg/m^2^) early-stage BC patients and healthy subjects, with no significant differential expression observed between normal weight early-stage BC patients and healthy subjects. Smoking, particularly water pipe smoking, revealed that circulating miR-155 and miR-451 were significantly upregulated and downregulated, respectively, in BC water pipe smokers as compared with non-smokers. In contrast to each other, miR-155 was significantly upregulated, while miR-451 was significantly downregulated in non-smoking BC patients vs. healthy subjects. Family history analysis revealed that miR-195 and miR-100 were significantly downregulated in early-stage BC patients with BC family history in contrast to those without family history. As compared with healthy subjects, miR-195 was significantly downregulated in those with BC family history, and miR-100 was significantly upregulated in early-stage BC patients without BC family history. In tumor size subgroups, miR-23a and miR-145 were significantly overexpressed in BC patients vs. healthy subjects, regardless of tumor size and also showed significant overexpression in association with tumor size. Early-stage BC patients with tumor size >2 and ≤5 cm (T2) had a significant increase in the level of expression of miR-23a and miR-145 as compared with patients with tumor size ≤2 cm (T1). As for the other subgroups, menopausal status, lymph node involvement, histological grade, cigarette smoking, alcohol intake, history of oral contraceptive pills, or hormonal replacement therapy use, there was no significant dysregulation in miRNA expression between the stratified groups.

### 2.4. Diagnostic Accuracy of miRNA in BC

**Figure 3:**
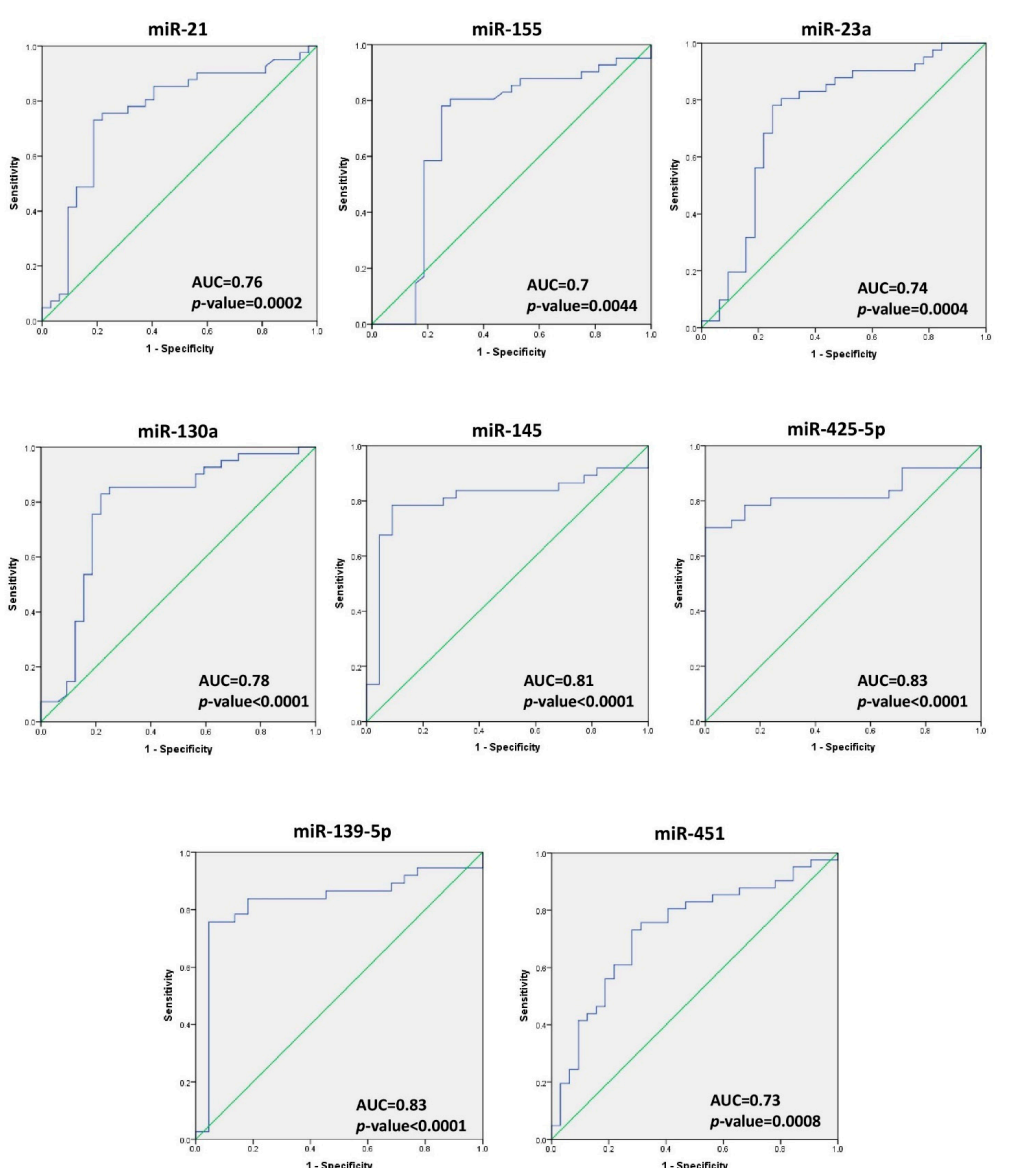
Diagnostic accuracy of miR-21, miR-130a, miR-155, miR-23a, miR-145, miR-425-5p, miR-139-5p, and miR-451 for BC detection in plasma. The ROC curve analysis separates between BC patients and healthy controls through individual miRNA. *p* < 0.05 indicates significance. For miR-21, miR-130a, miR-155, miR-23a, and miR-451, the number of tumor samples = 41 and the number of healthy samples = 32. For miR-145, miR-425-5p, and miR-139-5p, the number of tumor samples = 37 and the number of healthy subjects = 21.

The receiver operating characteristic (ROC) curve was used for the analysis of the performance of individual miRNAs for distinguishing between breast cancer patients and healthy controls. The ROC curve analysis revealed significant *p*-values for the area under the curve obtained for the eight significantly dysregulated miRNAs which were miR-21, miR-155, miR-23a, miR-130a, miR-145, miR-425-5p, miR-139-5p, and miR-451 (AUC = 0.76, 0.70, 0.74, 0.78, 0.81, 0.83, 0.83, and 0.73, respectively) (Figure 3). The optimal cut-off values, specificity, sensitivity, positive predicted value (PPV), negative predicted value (NPV), and diagnostic accuracy (DA) percentage for each miRNA were calculated (see Table 2). The microRNAs, miR-145 and miR-139-5p, scored the highest individual diagnostic accuracy (83% each) with an AUC of 0.81 ± 0.062 (*p* < 0.0001, 95% CI 0.686–0.928) for miR-145, and AUC of 0.83 ± 0.060 (*p* < 0.0001, 95% CI 0.710–0.946) for miR-139-5p; miR-130a scored the highest sensitivity (83%), while miR-425-5p had absolute specificity and NPV.

In order to analyze for correlations among expressions of the eight significantly dysregulated miRNAs, Spearman’s correlation coefficients were calculated (Table 3); miR-425-5p showed a significant and strong positive correlation with miR-145 (r = 0.920, *p*-value <0.01). Similarly, miR-21 had a significant and strong positive correlation with miR-23a and miR-130a (*r* = 0.909, *p* < 0.01 and *r* = 0.803, *p* < 0.01, respectively).

**Figure 4:**
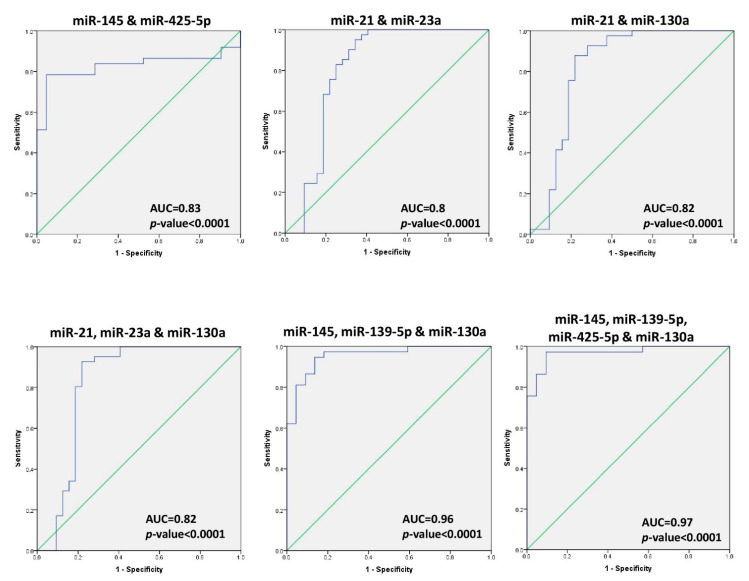
Diagnostic accuracy of combined miRNA for BC detection in plasma. Predicted probabilities were calculated using binary logistic regression to draw the ROC curve of the combined miRNAs. The ROC curve analysis separates BC patients and healthy controls through combinations of miRNAs. *p* < 0.05 indicates significance.

On the basis of the correlation test, the ROC analysis was conducted for the combined diagnostic ability of the miRNAs that have significant strong correlations. The optimal cut-off values, specificity, sensitivity, PPV, NPV, diagnostic accuracy, and 95% CI for each combination of miRNAs were calculated and presented in Table 2. The highest sensitivity, specificity, and diagnostic ability occurred for combinations of miR-23a, miR-21, and miR-130a. For other combinations based on increasing diagnostic accuracy of specific miRNAs see Figure 4. Results revealed that the combination of miR-145, miR-139-5p, and miR-130a scored higher diagnostic accuracy (92%), and the combination of miR-145, miR-139-5p, miR-130a, and miR-425-5p had the highest diagnostic accuracy overall (95%).

## 3. Discussion

Breast cancer has the highest mortality rate among Lebanese cancer patients, despite advanced treatment options and increased awareness and current screening guidelines [15]. Detection of BC in its early stages could drastically help to improve the disease outcome [16], hence, the need for minimally invasive and easily detected diagnostic biomarkers for BC diagnosis. Several studies have investigated the promising potential of microRNA molecules as diagnostic biomarkers for BC patients in tissues and blood circulation [8,14,17]. On the basis of the data generated here and literature review, miR-21, miR-155, miR-23a, miR-130a, miR-145, miR-451, miR-425-5p, miR-139-5p, miR-195, miR-125b, miR-100 and miR-182 are the miRNAs commonly studied in different ethnic groups to diagnose BC (Supplementary Material Table S2). The level of these 12 candidate microRNAs were quantified in plasma from early-stage BC patients, with the most common histotype and receptor profile in Lebanon (IDC and ER+/PR+) and compared to healthy controls to test for correlations between clinical and pathological data and their diagnostic ability.

The results show that miR-21, miR-155, and miR-23a are significantly overexpressed in plasma from early-stage BC patients as compared with healthy subjects, in accordance with other similar studies carried out in different ethnic groups [13,18,19,20,21]. As compared with miRNA expression in other sample types, miR-21, miR-155, and miR-23a expression in BC tissue samples show a similar trend to that documented in patient plasma samples in this study [22,23]. Similar to upregulation of serum levels of miR-21 and miR-155 upregulated in Chinese ethnic groups [24], patients in this study confirmed those findings. In accordance with their overexpression, these miRNA molecules play an oncogenic role in BC development. It has been shown that miR-21 overexpression promoted BC progression and chemoresistance via TGF-β/miR-21/phosphatase and tensin homolog (PTEN) signaling axis suppression [25]; miR-21 also targeted tissue inhibitor of matrix metalloproteinases 3 (TIMP3), programmed cell death receptor 4 (PDCD4), tropomyosin 1 (TPM1), and reversion-inducing cysteine-rich protein with kazal motifs (RECK) mRNA, therefore, enhanced cell invasion, tumor metastasis, and angiogenesis [26]. In addition, miR-155 has been shown to promote proliferation and migration of BC cells through downregulation of the suppressor of cytokine signaling 1 (SOCS1) and upregulation of the matrix metallopeptidase 16 (MMP16) [27] and miR-23a has been shown to enhance BC progression by directly activating Forkhead Box M1 (FOXM1) and histidine-rich glycoprotein (HRG) at the RNA level [23].

Expression of miR-130a, miR-145, and miR-451 in plasma from early-stage BC Lebanese patients was not similar to that in other ethnic groups. The microRNAs, miR-145 and miR-130a, were upregulated in Lebanese patients, yet studies in different ethnic groups have revealed those miRNAs to be downregulated in tissue and plasma samples [13,19,28,29,30]. Additionally, miR-130a has been shown to inhibit cell proliferation, invasion and migration by targeting Ras analog in brain mRNA (RAB5A) [30], reducing expression of FOS-like antigen 1 (FOSL-1) and suppressing inhibition of zonula occludins-1 (ZO-1) [31]. The role of miR-145 in BC has been shown to suppress cancer cell migration by targeting FSCN and inhibiting epithelial-mesenchymal transition [32]. It inhibits transforming growth factor (TGF-β1) protein expression which contributes to tumor formation [28]. In this study, miR-451 is downregulated in plasma of Lebanese patients as compared with its upregulation in plasma of a Chinese ethnic group [19,33]. Studies have shown that miR-451 is involved in tumor initiation and progression [34], and, it has been reported to play a role in influencing resistance of paclitaxel-resistant BC cell lines [35,36]. As for the expression of miR-451 in tissue samples, there are contradicting results regarding its mode of regulating dysregulation [19,36]. The contradictory results between this study and other studies are potentially due to different histopathological types of BC, different receptor statuses, or different pathological stages of BC patients participated.

As for miR-425-5p and miR-139-5p upregulated in plasma of BC patients, no previous studies have evaluated the mode of dysregulation in plasma of BC patients. One study reported that miR-425-5p was upregulated while miR-139-5p was downregulated in sera of Caucasian ER-positive early BC patients [37], and miR-425-5p has been reported to play an oncogenic role in breast cancer by promoting cell invasion and migration via PTEN [38]. According to Krishnan et al. [39], miR-139-5p prevents cell migration and metastasis by disrupting the TGFβ, Wnt, Rho, and MAPK/PI3K signaling cascades.

In this study, the abundance of miR-195, miR-125b, miR-100, and miR-182 in plasma displayed non-remarkable differences between BC patients and healthy controls. Other studies have also reported this non-significant dysregulation in expression of miR-182 and miR-195 in the plasma of BC patients [13,40]. Contradictory results for miR-195 expression in plasma samples have been reported with some showing downregulation in plasma samples [41,42], and a study of Saudi women with triple negative BC uncovering an increase in plasma [43]. Moreover, according to the literature, miR-125b has been shown to be abundant in plasma from Spanish BC patients and in serum of Mexican BC patients [18,29]. Regarding miR-100, it has been shown to be significantly under expressed in BC tissues as compared with adjacent normal breast among Iranian women [44]. Hence, the level of expression of each miRNA differs among ethnic groups.

Our results highlight the important and significant associations of miRNA biomarkers with early-stage BC between dysregulated miRNA and different clinicopathological BC subgroups. BC patients with normal weight showed overexpression of miR-451 as compared with levels in the obese/overweight group. Since waterpipe smoking is endemic in this region, finding a significant overexpression of miR-155 and an under expression of miR-451 in the plasma of the subgroup of waterpipe smoking patients as compared with non-smokers is worthwhile noting, in spite of the small sample size. Dividing patients into subgroups uncovered that miR-195 and miR-100 levels differentiated between BC patients with positive BC family history from those without. Despite the insignificant deregulation of miR-195 and miR-100 in all BC patients, their significant differential expression upon subgrouping based on BC family history might reflect other findings in which familial BC patients or sporadic BC patients might have participated. Although miR-21 overexpression has been reported to be associated with clinical characteristics of BC patients, including tumor grade and lymph node metastasis [45], it didn’t show any relationship with any of the assigned subgroups in this study. Interestingly, miR-145 and miR-23a tend to be overexpressed in early-stage BC with larger tumor size. Similar observations were reported by Li et al. for miR-23a [13].

The ROC curve analysis of individual miRNA revealed that miR-145 and miR-139-5p achieved the highest diagnostic accuracy (83% each) among all miRNAs. Better diagnostic abilities emerged when combining different miRNAs (see Table 2). The combination of four miRNAs (miR-145, miR-139-5p, miR-130a, and miR-425-5p) showed the highest and best sensitivity, specificity, and diagnostic accuracy (97%, 91%, and 95%, respectively) of all individual and combined miRNAs for detecting early-stage BC patients. Another study on 170 breast Chinese breast cancer patients and 100 healthy subjects revealed that the combination of plasma miR-145 and miR-451 provided a useful biomarker for early-stage breast cancer (ductal in situ carcinoma) detection [19].

Although this study has some limitations such as small sample size and focus on ER-positive BC samples, it demonstrated distinctive expression patterns of miRNAs, suggesting their value as non-invasive diagnostic molecular biomarkers for early-stage BC patients. Dysregulated miRNAs evaluation in larger experimental groups, particularly miR-145, is necessary. This is due to the fact that the latter is associated with larger tumor size, has the highest individual diagnostic accuracy, and is included in the combination of mRNAs which scored the highest diagnostic accuracy. Whereas a signature of dysregulated circulating miRNAs has a promising potential role in diagnosing early-stage BC cases, some discrepancy with previous reports exists. This could be due to differences in sample size, in ethnic origin, and age groups chosen, as well as differences in BC molecular and/or histopathological subtypes.

## 4. Materials and Methods

### 4.1. Specimen Collection

This study was approved by the Institutional Review Board at the American University of Beirut (IRB number IM.RN.02; date of approval: 16 March 2015) and it was in accordance with the Helsinki Declaration. Written informed consent was obtained from all participants. Whole blood samples were collected from a total of 73 Lebanese women who participated in this study. Forty-one participants were BC patients newly diagnosed between September 2012 and May 2014 at the American University of Beirut Medical Center (AUBMC) and 32 participants were healthy female controls. All recruited participants were female and of Lebanese nationality. Patient clinicopathological characteristics were tabulated, including age at diagnosis, menopausal status, tumor grade, stage, and human epidermal growth factor receptor 2 (HER2) status.

### 4.2. Total RNA Extraction

Peripheral whole blood was withdrawn and immediately centrifuged at a speed of 10,000 rpm for 10 min at 4 °C, and the isolated plasma was stored at −80 °C until analysis. Then, total RNA was extracted using a Plasma/Serum Circulating and Exosomal RNA Purification Kit (Norgen Biotek Corp., Thorold, ON, Canada), following the manufacturer’s protocol. RNA concentration and quality were assessed using DeNovix DS-11 FX spectrophotometer (Delaware, DE, USA). Extracted RNA samples were stored at −80 °C until analysis.

### 4.3. cDNA Synthesis

After extraction of plasma total RNA, the complementary DNA (cDNA) of the microRNAs of interest were synthesized from 10 ng of extracted RNA using a TaqMan^®^ MicroRNA Reverse Transcription Kit (Applied Biosystems, Waltham, MA, USA), following manufacturer protocols. The following human primers (hsa-miR-16, hsa-miR-23a, hsa-miR-155, hsa-miR-23a, hsa-miR-130a, hsa-miR-145, hsa-miR-425-5p, hsa-miR-139-5p, hsa-miR-451, hsa-miR-195, hsa-miR-125b, hsa-miR-100, and hsa-miR-182) and their corresponding probes were purchased as part of the validated TaqMan^®^ microRNA Assays Kit (Applied Biosystems, Waltham, MA, USA). The synthesized cDNA was diluted, and then stored at −20 °C until analysis.

### 4.4. Quantitative Real-Time Polymerase Chain Reaction (RT-qPCR) Analysis

To detect miRNA expression, RT-qPCR (BioRad CFX384 Real Time System, C1000 thermal cycler, Hercules, CA, USA) was carried out in duplicate for each sample using 2x TaqMan^®^ Universal Master Mix with no Amperase Uracil N-Glycosylase (UNG) (Applied Biosystems, Waltham, MA, USA) including a ‘no template’ control for each miRNA, following manufacturer protocols. The cycling conditions were 10 min at 95 °C, then, 40 cycles of 15 s at 95 °C (denaturing step) and 60 s at 60 °C (annealing and extension steps). The miR-16 showed the same expression in plasma from tumor and healthy samples with a coefficient of variation (CV) = 8.7% and was used as an endogenous control. Similarly, miRNA molecules of interest showed constant expression in all healthy subjects. Therefore, the relative expression of selected miRNA was normalized to the chosen endogenous control and was compared to healthy controls. The relative fold change of expression was calculated using the ∆∆Ct equation.

### 4.5. Statistical Analysis

Statistical analysis was performed using GraphPad Prism 6 software (San Diego, CA, USA) and Statistical Package for Social Sciences (SPSS) version 22 (Armonk, NY, USA). The Shapiro–Wilk test was used to test for normality distribution after which expression data were compared with non-parametric tests. The significance of the fold change expression of miRNA in BC as compared with one was determined using one sample non-parametric test Wilcoxon’s signed-rank test. The Mann–Whitney U and Kruskal–Wallis tests were applied to detect any significant dysregulation of the miRNA within two or more clinicopathological subgroups. *p*-values <0.05 were considered to be statistically significant. The ∆Ct values were utilized to plot receiver operating characteristic (ROC) curves of individual miRNA, in order to determine the diagnostic accuracy and parameters of single miRNA molecules tested. According to the fold change of expression values, the correlation between the expression of each pair of miRNAs was assessed by calculating spearman’s correlation coefficient. Binary logistic regression was utilized to draw the ROC curve of the combined miRNA. Youden’s index was calculated to identify the best cut-off, sensitivity, and specificity for each miRNA.

## 5. Conclusions

This study is the first to investigate the potential of plasma microRNAs for detecting early stage of IDC BC with ER-positive and PR-positive status among Lebanese women. Importantly, significantly dysregulated miRNAs (miR-21, miR-155, miR-130a, miR-451, miR-23a, miR-425-5p, miR-145, miR-425, and miR-139-5p) in early-stage BC as compared with healthy controls emerged. Furthermore, a panel of four miRNAs (miR-145, miR-139-5p, miR-425-5p, and miR-130a) showed high accuracy for detecting early-stage BC cases. These data provide the basis for future research studies, which should include larger sample sizes with diverse receptor profiles and breast cancer pathological subtypes to validate dysregulated circulating miRNA molecules, particularly the correlation between the expression of each pair of miRNAs in patients with early-stage BC.

## Figures and Tables

**Table 1:** Clinical and pathological characteristics of the Lebanese breast cancer patients participating in this study. Normal weight BC patients have BMI values >20 and <24.9 kg/m^2^, while overweight/obese BC patients have BMI values >25 kg/m^2^. The American Joint Committee on Cancer (AJCC) TNM staging system was used to indicate tumor size, lymph node involvement, and distant metastasis.

Clinicopathological Characteristics		Number	Percentage (%)
Total number of breast cancer (BC) cases		41	100
Age (years)	Mean ± SD	53 ± 11.88	
	Range	30–84	
Menopausal status	Premenopausal	17	41.5
	Postmenopausal	22	53.7
	Unknown	2	4.9
Age at menarche (years)	≤12	17	41.5
	13	12	29.3
	≥14	10	24.4
	Unknown	2	4.9
BMI ^1^	Normal weight	20	48.8
	Overweight/obese	18	44.0
	Unknown	3	7.3
Family history of BC	Yes	17	41.5
	No	22	53.6
	Unknown	2	4.9
Cigarette smoking	Yes	12	29.3
	No	26	63.4
	Unknown	3	7.3
Waterpipe smoking	Yes	5	12.2
	No	33	80.5
	Unknown	3	7.3
Alcohol intake	Yes	10	24.4
	No	28	68.3
	Unknown	3	7.3
History of OCP Use ^2^	Yes	23	56.1
	No	16	39.0
	Unknown	2	4.9
History of HRT use ^3^	Yes	8	19.5
	No	31	75.6
	Unknown	2	4.9
HER-2 status	Positive	26	63.4
	Equivocal	12	29.3
	Negative	3	7.3
Histological grade	1	17	41.5
	2	16	39.0
	3	7	17.1
	Unknown	1	2.4
Tumor size	Tumor size (T1) ≤2 cm	27	65.9
	Tumor size (T2) >2 cm but ≤5 cm	13	31.7
	Tumor size (T3) >5 cm	1	2.4
Lymph node involvement	No	25	61.0
	Yes	15	36.6
	Unknown	1	2.4
Distant metastasis	Yes	0	0
	No	41	100

^1.^ BMI, body mass index; ^2.^ OCP, oral contraceptive pills; ^3.^ HRT: hormone replacement therapy.

**Table 2:** Diagnostic parameters to evaluate the early-stage BC diagnostic ability of individual and combined studied miRNA. The ROC curve reports the AUC of each miRNA. Youden’s index was calculated to determine the cut-off value, sensitivity, specificity, PPV, NPV, and DA of each miRNA. Predicted probabilities were calculated and utilized in case of combined miRNA molecules.

miRNA	AUC ^a.^	*p*-Value	SE ^b.^	95% CI ^c.^	Youden’s Index	Cut-Off	Sensitivity (%)	Specificity (%)	PPV (%) ^d.^	NPV (%) ^e.^	DA (%) ^f.^
miR-21	0.76	0.0002	0.06	0.640–0.876	0.54	4.46	73	81	76	78	77
miR-155	0.70	0.0044	0.07	0.559–0.832	0.53	10.54	78	75	78	75	77
miR-23a	0.74	0.0004	0.063	0.619–0.866	0.53	11.69	78	75	88	44	68
miR-130a	0.78	<0.0001	0.060	0.660–0.896	0.61	10.18	83	78	83	78	81
miR-145	0.81	<0.0001	0.062	0.686–0.928	0.69	9.09	78	91	78	91	83
miR-425-5p	0.83	<0.0001	0.056	0.716–0.936	0.70	7.96	70	100	70	100	81
miR-139-5p	0.83	<0.0001	0.060	0.710–0.946	0.71	8.54	76	96	76	95	83
miR-451	0.73	0.0008	0.060	0.613–0.849	0.45	2.5	73	72	73	72	73
miR-145 + miR-425-5p	0.83	<0.0001	0.057	0.716–0.940	0.74	0.62	78	95	78	95	84
miR-21 + miR-23a	0.80	<0.0001	0.061	0.684–0.924	0.61	0.38	95	66	95	66	82
miR-21 + miR-130a	0.82	<0.0001	0.058	0.710–0.937	0.659	0.42	88	78	88	78	84
miR-21 + miR-23a + miR-130a	0.82	<0.0001	0.061	0.702–0.940	0.71	0.46	93	78	93	78	86
miR-145 + miR-139-5p + miR-130a	0.96	<0.0001	0.026	0.905–1.000	0.81	0.39	95	86	95	86	92
miR-145 + miR-139-5p + miR-130a + miR-425-5p	0.97	<0.0001	0.020	0.929–1.000	0.88	0.36	97	91	97	90	95

^a.^ AUC, area under the curve; ^b.^ SE, standard error; ^c.^ CI, confidence interval; ^d.^ PPV, positive predicted value; ^e.^ NPV, negative predicted value; ^f.^ DA, diagnostic accuracy.

**Table 3:** Spearman’s correlation coefficients between each pair of miRNAs. * denotes significant correlation coefficient (*p*-value < 0.05).

	miR-21	miR-155	miR-23a	miR-130a	miR-145	miR-425-5p	miR-139-5p	miR-451
miR-21	1.00	0.752 *	0.909 *	0.803 *	0.682 *	0.596 *	0.582 *	0.12
miR-155		1.00	0.695 *	0.737 *	0.672 *	0.607 *	0.483 *	−0.16
miR-23a			1.00	0.781 *	0.676 *	0.626 *	0.56 *	−0.13
miR-130a				1.00	0.654 *	0.59 *	0.506 *	−0.03
miR-145					1.00	0.920 *	0.713 *	−0.20
miR-425-5p						1.00	0.726 *	−0.22
miR-139-5p							1.00	−0.19
miR-451								1.00

## Data Availability

The datasets generated during the current study are not publicly available due to patient privacy but are available from the corresponding author on reasonable request.

## Supplementary Materials

The following are available online at https://www.mdpi.com/article/10.3390/ijms22116121/s1, Figure S1: Fold change of expression for the non-significantly dysregulated miRNA in the plasma of Lebanese women with early-stage BC as compared with healthy controls, Table S1: Fold change of expression for the studied miRNA in plasma of Lebanese early stage BC patients sub-grouped into different clinical and histopathological presentations, Table S2: Comparison between the mode of deregulation of miR-21, miR-155, miR-23a, miR-130a, miR-145, miR-451, miR-425-5p, and miR-139-5p in our study and in the literature.
