# Supplementary material for: A Signature of Four Circulating microRNAs as Potential Biomarkers for Diagnosing Early-Stage Breast Cancer
Source: Int J Mol Sci. 2021 Jun 6;22(11):6121. doi: 10.3390/ijms22116121 (PMC8200990; doi:10.3390/ijms22116121)
Supplement: Supplementary file 1 [file ijms-22-06121-s001.zip › ijms-1216689-SI.pdf]

Mode of Dysregulation	↑		↑		↑		↑		↑		↑		↑		↓		▬		▬		▬		▬	
Subgroups Characteristics	**miR-21**		**miR-155**		**miR-23a**		**miR-130a**		**miR-145**		**miR-425-5p**		**miR-139-5p**		**miR-451**		**miR-195**		**miR-125b**		**miR-100**		**miR-182**	
	mean	*p*-value	mean	*p*-value	mean	*p*-value	mean	*p*-value	*mean*	*p*-value	*mean*	*p*-value	*mean*	*p*-value	mean	*p*-value	mean	*p*-value	*mean*	*p*-value	*mean*	*p*-value	*mean*	*p*-value
**Menopausal Status**<br>Premenopausal<br>Postmenopausal	3.517<br>3.708	0.883	1.802<br>0.779	0.282	2.689<br>3.034	0.479	9.118<br>10.89	0.435	11.59<br>11.64	0.433	3.900<br>5.089	0.163	7.468<br>10.34	0.257	0.914<br>0.696	0.082	4.947<br>5.720	0.979	1.215<br>1.261	0.922	1.821<br>1.279	0.513	0.914<br>0.696	0.245
**BMI**<br>Normal weight<br>Overweight/Obese	3.459<br>3.909	0.961	1.662<br>0.751	0.286	2.490<br>3.440	0.136	10.40<br>10.46	0.518	12.41<br>1.47	0.831	4.337<br>5.045	0.438	8.505<br>10.19	0.600	0.869<br>0.635	**0.027**	4.704<br>6.395	0.722	1.137<br>1.408	0.737	1.651<br>1.467	0.750	1.603<br>1.779	0.678
**Family Hx of BC**<br>No<br>Yes	3.922<br>3.240	0.295	1.145<br>0.636	**0.010**	3.295<br>2.352	0.140	11.82<br>6.365	0.088	13.58<br>9.312	0.116	5.446<br>3.550	0.068	11.22<br>6.603	0.105	0.708<br>0.899	0.264	5.924<br>4.682	0.161	1.168<br>11.22	0.293	1.950<br>1.039	**0.028**	1.895<br>1.362	0.101
**Cigarette Smoking**<br>No<br>Yes	3.79<br>3.218	0.648	1.349<br>0.748	0.211	3.400<br>1.892	0.056	10.30<br>8.209	0.172	11.70<br>12.13	0.508	5.019<br>3.911	0.374	9.783<br>8.186	0.741	0.721<br>0.930	0.051	5.668<br>4.606	0.551	1.271<br>1.225	0.742	1.664<br>1.228	0.144	1.785<br>1.451	0.569
**Waterpipe Smoking**<br>No<br>Yes	3.526<br>4.620	0.350	1.187<br>0.868	0.776	2.665<br>4.631	**0.028**	9.327<br>11.71	0.068	11.90<br>11.42	0.372	4.474<br>5.963	0.240	9.268<br>9.461	0.559	0.831<br>0.529	**0.005**	5.072<br>7.889	0.252	1.290<br>1.055	0.665	1.473<br>1.949	0.115	1.702<br>1.505	0.717
**Alcohol Intake**<br>No<br>Yes	3.943<br>3.000	0.423	1.319<br>0.717	0.134	3.345<br>1.888	0.134	10.21<br>8.251	0.327	11.51<br>12.68	0.699	4.971<br>3.925	0.524	9.655<br>8.359	0.721	0.818<br>0.726	0.929	6.076<br>3.888	0.286	1.339<br>1.045	0.186	1.556<br>1.456	0.782	1.758<br>1.485	0.647
**History of OCP Use**<br>No<br>Yes	3.508<br>3.706	0.905	0.806<br>1.516	0.298	3.207<br>2.659	0.272	9.406<br>9.472	0.772	14.32<br>9.775	0.275	4.483<br>4.483	0.566	11.06<br>7.759	0.486	0.757<br>0.815	0.587	4.400<br>6.067	0.431	1.234<br>1.246	0.440	1.681<br>1.417	0.892	1.617<br>1.660	0.755
**History of HRT Use**<br>No<br>Yes	3.429<br>4.383	0.480	1.217<br>1.256	0.938	2.752<br>3.394	0.531	8.606<br>12.70	0.445	12.22<br>9.024	0.805	4.880<br>3.267	0.454	9.063<br>9.244	0.631	0.776<br>0.849	0.665	5.348<br>5.518	0.985	1.286<br>1.047	0.783	1.435<br>1.871	0.691	1.684<br>1.474	0.807
**Histological Grade**<br>G1<br>G2<br>G3	3.820<br>3.511<br>2.981	0.878	1.633<br>0.999<br>0.770	0.543	2.957<br>2.798<br>2.948	0.967	9.639<br>11.96<br>7.326	0.870	11.42<br>12.75<br>10.67	0.944	4.585<br>4.053<br>5.455	0.703	8.000<br>11.40<br>6.777	0.697	0.785<br>0.797<br>0.724	0.889	5.584<br>5.566<br>4.433	0.939	1.148<br>1.680<br>0.744	0.123	1.523<br>1.770<br>0.990	0.916	1.812<br>1.611<br>1.298	0.858
**Tumor Size**<br>T1<br>T2	2.996<br>4.570	0.053	0.908<br>1.815	0.591	2.650<br>3.335	0.266	7.492<br>15.29	0.066	8.983<br>17.11	**0.023**	3.453<br>6.912	0.087	8.960<br>9.382	0.648	0.818<br>0.679	0.484	4.137<br>7.566	**0.013**	1.368<br>0.977	0.427	1.428<br>1.729	0.295	1.326<br>2.363	0.139
**Lymph-node Involvement**<br>No<br>Yes	3.878<br>3.167	0.387	1.471<br>0.824	0.788	3.135<br>2.625	0.539	8.461<br>13.62	0.852	12.20<br>11.22	0.530	4.839<br>4.211	0.401	9.387<br>9.066	0.355	0.732<br>0.848	0.451	5.772<br>4.870	0.572	1.215<br>1.187	0.679	1.685<br>1.153	0.287	1.643<br>1.731	0.605

Supplementary Table S1. Fold change of expression of the studied miRNA in plasma of Lebanese early stage BC patients sub-grouped into different clinical and histopathological presentations. *p*-value was calculated using Mann-Whitney *U* or Kruskal-Wallis nonparametric tests. Kruskal-Wallis test was used in case of having more than 2 groups in one clinicopathological category such as histological grade. Highlighted values point to significant *p*-value (*p*-value < 0.05). – denotes non-significantly dysregulated miRNA.

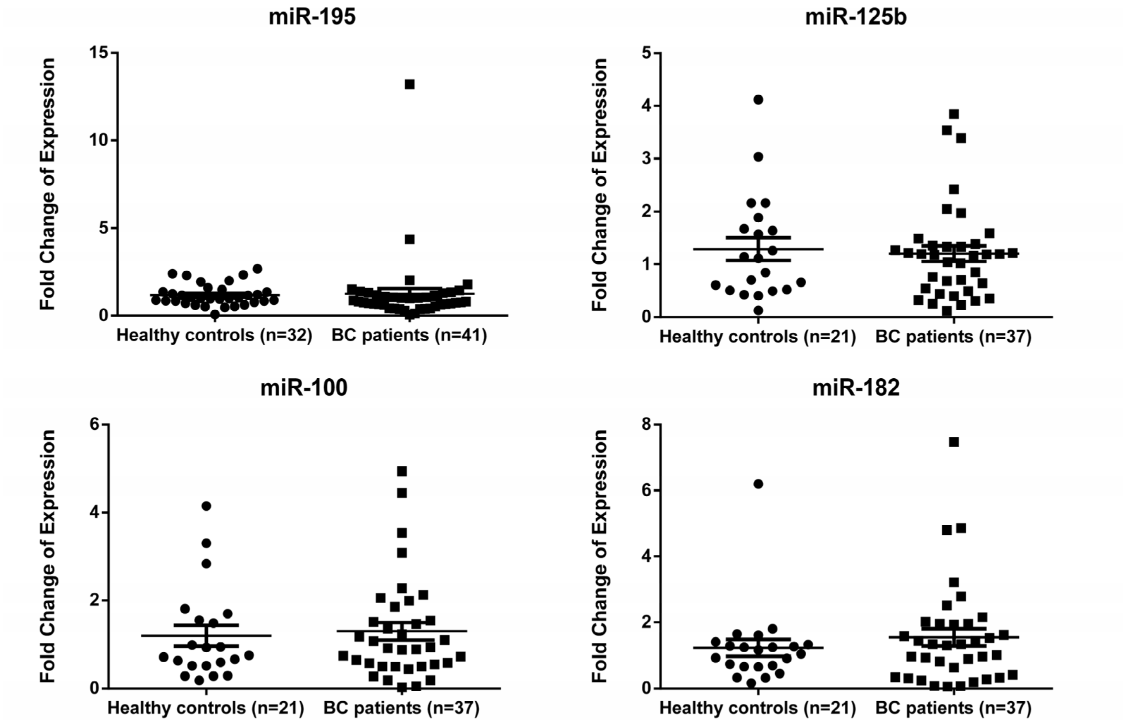

Supplementary Figure S1. Fold change of expression of the non-significantly dysregulated miRNA in the plasma of Lebanese women with early stage BC as compared to healthy controls. The plots represent the mean (middle line) and the standard error of mean (error bars). *p*-value > 0.05 according to Wilcoxon's signed-rank test.
